# Comparison of personality and psychopathology in patients with Fabry disease and patients with end-stage renal disease: a preliminary study

**DOI:** 10.3389/fpsyt.2025.1460260

**Published:** 2025-02-27

**Authors:** Concetta De Pasquale, Maria Luisa Pistorio, Massimiliano Veroux, Tania Moretta, Margherita Stefania Rodolico, Ines Paola Monte, Noemi Barbagallo, Luca Zanoli, Denise Cristiana Faro, Alessia Giaquinta, Martina Maria Giambra, Pierfrancesco Veroux

**Affiliations:** ^1^ Department of Educational Science, University of Catania, Catania, Italy; ^2^ Vascular Surgery and Organ Transplant Unit, Department of General Surgery and Medical-Surgical Specialties, University Hospital of Catania, Catania, Italy; ^3^ Organ Transplant Unit, Department of Surgical and Medical Sciences and Advanced Technologies, University Hospital of Catania, Catania, Italy; ^4^ Department of General Psychology, University of Padova, Padova, Italy; ^5^ Consiglio Nazionale delle Ricerche (C.N.R.) Institute for Biomedical Research and Innovation-IRIB, Section of Catania, Catania, Italy; ^6^ Department of General Surgery and Medical-Surgical Specialties, University Hospital of Catania, Catania, Italy; ^7^ Department of Clinical and Experimental Medicine, University of Catania, Catania, Italy

**Keywords:** clinical psychology, personality, psychopathology, Fabry disease, end stage renal disease

## Abstract

**Background:**

The present study aimed to investigate personality characteristics and psychopathological symptoms in patients with Fabry disease (FD) vs a group of individuals with end-stage renal disease (ESRD).

**Methods:**

A total of 36 patients, equally divided into patients with FD and patients with ESRD (control group), were administered the following tools: the Millon Clinical Multiaxial Inventory III (MCMI-III) to evaluate personality psychopathology and the Symptom Checklist-90-R (SCL-90-R) to assess symptoms of psychopathology.

**Results:**

Significantly higher levels of Schizoid, Depressive, and Negativistic personality traits emerged in FD patients. Moreover, statistically significant differences in Anxiety, Interpersonal Sensitivity, Obsessive-Compulsive, Depression, Somatization, and Psychoticism dimensions of the SCL-90-R were found, with higher levels of each dimension in patients with FD than ESRD.

**Conclusions:**

The literature, albeit limited, highlights how patients with FD are at higher risk of developing psychological distress and psychopathology than patients presenting other chronic diseases such as ESRD. Using psychological therapies together with standard treatments for FD can promote condition acceptance, reduce emotional burden, and relieve psychopathological symptoms in FD patients.

## 1 Introduction

Anderson-Fabry disease (FD) is a rare condition caused by lysosomal accumulation due to a deficiency of the alpha-galactosidase A enzyme. This leads to the buildup of complex glycolipids, particularly globotriaosylceramide (Gb3), in visceral tissues and vascular endothelium throughout the body. This systemic accumulation causes significant damage to the kidneys, heart, and central nervous system, leading to impaired quality of life and severely compromised social functioning (1, 2).

Regarding routine activities, individuals with FD struggle to perform normal daily tasks and face challenges in social functioning (3–6). Patients with FD face substantial challenges in their daily lives, including physical symptoms such as chronic pain, fatigue, and organ dysfunction, alongside psychological stressors such as isolation, fear of judgment, and difficulties in interpersonal relationships (7–9). The psychosocial burden of FD is further underscored by research indicating a high prevalence of psychopathological symptoms, including depression, anxiety, and cognitive dysfunction, thus they are also considered at higher risk of developing psychopathological symptoms, potentially progressing to undiagnosed psychiatric disorders. Feelings of loneliness and isolation (33.3%), anxiety (66.7%), and depression, have recently been reported together with problems in interpersonal relationships, and fear of judgment from others (66.7%) in a sample of 106 patients with FD (5). A systematic review by Bolsover et al. (8) investigated factors predominantly associated with depression in FD patients. The study revealed a prevalence of depression ranging from 15% to 62%, with neuropathic pain being the most common associated factor, influencing social and adaptive functioning. Interestingly, gender norms for depression in FD do not follow the typical pattern, with males being more frequently affected than females (8, 10). Moreover, Körver et al. highlighted depressive symptoms in 81 FD patients, revealing that those using “avoidance and brooding” coping behavior had more depressive symptoms. This underscores the importance of functional coping strategies in alleviating depressive symptoms in FD patients (11).

### 1.1 Personality and psychopathology in FD

Segal et al. (12) studied 16 FD patients and found that 10 of them met the diagnostic criteria for Axis I (depressive disorders) or II (borderline personality disorders) of the Diagnostic and Statistical Manual of Mental Disorders - fourth edition (DSM-IV), along with cognitive issues, emphasizing the importance of psychopathological assessment in these patients (12). In this context, the assessment of personality traits and psychopathological symptoms in FD patients becomes crucial. Personality characteristics play a significant role in how individuals cope with chronic illness and its associated stressors, influencing treatment adherence and overall quality of life. Previous studies have identified maladaptive personality traits, such as depressive and schizoid tendencies, as well as elevated psychopathological symptoms in FD patients. These traits may be linked to the unique and pervasive nature of FD, which often disrupts multiple domains of life due to its systemic and progressive course.

In a study by Laney et al. high rates of antisocial personality traits and aggressive behavior were found in 30 patients with FD (10).

The assessment of psychopathological symptoms in populations with chronic illness is critical due to the significant overlap between physical and mental health, the potential for one to exacerbate the other, and the complex relationship between physical disease and psychological distress.

The Biopsychosocial Model (3) posits that health and illness should be understood in terms of the interaction between biological, psychological, and social factors, rather than simply focusing on biological aspects alone. This model provides a foundation for understanding how chronic illness impacts mental health (3).

Seligman’s Learned Helplessness Theory (1975) suggests that individuals with chronic illness may develop a sense of powerlessness and loss of control over their situation, which can lead to depression. If patients repeatedly experience situations where they feel their efforts to improve their health are futile, they may exhibit depressive symptoms, including low motivation, hopelessness, and withdrawal from treatment. This theory highlights the importance of assessing depressive symptoms and the individual’s coping mechanisms in managing chronic illness (13).

There is significant evidence showing that individuals with chronic illness are at a higher risk for psychopathological symptoms, particularly depression and anxiety. Chronic illnesses often come with prolonged stress, uncertainty, pain, and lifestyle changes, all of which can trigger or exacerbate mental health disorders (7–9, 11).

Given the high rates of psychopathological symptoms in these populations, systematic assessment is essential to ensure that mental health needs are adequately addressed alongside the physical health concerns.

Crosbie et al. (14) highlighted, in 28 patients with FD, aspects indicative of psychological distress (depressive reactions and somatic disorders) with significantly higher scores in seven clinical scales of the Minnesota Muliphasic Personality Inventory (MMPI-2): Hypocondriasis, Depression, Hysteria, Psychopathic Deviate, Paranoia, Psychasthenia, and Schizophrenia, compared with normative references. Additionally, these patients showed higher scores than those with Gaucher disease, chronic heart disease, and chronic pain (14).

### 1.2 The Five-Factor Model of personality

The link between personality and psychopathology has been widely explored in psychological research, and numerous studies have provided strong evidence regarding the relationship between personality traits and the risk of developing psychopathological disorders.

This link has been theorized in various models, including the Five-Factor Model (FFM) of personality, which identifies five core personality traits: neuroticism, extraversion, conscientiousness, openness to experience, and agreeableness. Each trait has been shown to be linked to specific psychopathological outcomes, and several theories have attempted to understand how these traits influence mental health. For example, neuroticism is one of the traits most strongly correlated with psychopathological disorders, particularly internalizing disorders, such as depression, anxiety, and post-traumatic stress disorder. People with high levels of neuroticism tend to be emotionally unstable, susceptible to stress, and to experience worry and anxiety. This trait is associated with increased emotional reactivity and a tendency to overreact to stressful events, a factor that contributes to the development of psychopathological symptoms (15, 16).

Agreeableness, which includes cooperation, trust and empathy, is associated with good mental health and a lower likelihood of developing psychopathological disorders. However, low levels of agreeableness have been correlated with disorders such as antisocial personality disorder, narcissistic disorder and borderline personality disorder, where difficulties in intimacy and emotional regulation can lead to aggressive and interpersonal dysfunctional behaviors (17, 18).

Impulsive or aggressive traits, such as those related to borderline personality disorder or antisocial personality disorder, may be linked to externalizing symptoms such as hostility, aggression and behavioral disorders. People with high levels of impulsivity tend to have difficulty controlling aggressive behaviors (19).

Perfectionism, which can be associated with traits of obsessive-compulsive personality disorder, is a trait that is linked to psychopathological symptoms such as obsessiveness and compulsions. People with high levels of perfectionism and mental rigidity tend to be excessively concerned with details and performance, increasing the risk of developing compulsive behaviors and obsessive symptoms (20).

Research highlights how neurotic, impulsive, relational and perfectionist traits can be related to specific psychopathological symptoms such as anxiety, depression, obsessive-compulsive disorders and externalizing behaviors. These links are essential to understanding how personality affects mental health and how the assessment of personality traits can help predict psychopathological symptoms in clinical contexts.

Based on these premises, and since personality characteristics mighty play an important role in characterizing coping strategies and patients’ stress responses to their chronic condition, our preliminary study firstly aimed to explore whether patients with FD are characterized by specific personality patterns. Assessing personality in FD can be important to understand how patients with FD perceive, interpret and evaluate the meaning of their chronic disease.

Our preliminary study also intended to evaluate any specific psychopathological symptoms of FD patients, since their presence can influence the course of patients’ medical conditions and quality of life. Studies such as Huprich (21) on depressive personality traits and their connection to chronic hopelessness, as well as Bleidorn et al. (22) discussing life events and personality changes, provide further context. Additionally, the biopsychosocial framework by Engel (3) and studies on maladaptive personality traits in chronic illness such as that by Davis et al. (23) offer fundamental support for these associations. Based on existing literature, depressive personality traits are most prominently linked to outcomes such as chronic depression and anxiety, characterized by persistent hopelessness and withdrawal. Similarly, schizoid traits, reflecting detachment and restricted affect, are often associated with psychoticism and interpersonal sensitivity. Negativistic (passive-aggressive) traits can predict interpersonal conflict and hostility. Notably, Mroczek et al. (24) provided a comprehensive review of neuropsychiatric symptoms and behavioral manifestations in FD patients, highlighting avoidant and antisocial personality traits as particularly prevalent.

### 1.3 Why the comparison between FD and ESRD?

In the study of Crosbie et al. (14) the personality of patients with FD is evaluated in comparison with patients with other chronic diseases: chronic heart disease, Gaucher disease and chronic pain. We chose to compare FD patients with those presenting a chronic renal failure disease, namely End-Stage Renal Disease (ESRD), as impaired renal function is often present in FD as well. Furthermore, patients with ESRD present severe clinical psychological and psychopathological distress due to dialysis therapy with negative repercussions on their quality of life (25). Similarly, ESRD represents another chronic condition associated with significant psychological and emotional burden. Patients with ESRD often experience distress related to dialysis therapy, lifestyle limitations, and a sense of dependency on medical interventions. Research has shown that ESRD patients frequently exhibit symptoms of depression, anxiety, and interpersonal sensitivity, which are linked to the demands of managing their condition (25, 26).

In particular, patients with FD were compared with patients with ESRD for several important reasons:

Fabry disease often leads to renal impairment, which can progress to chronic kidney failure. This makes ESRD patients a suitable control group for evaluating the impact of FD compared with another severe renal conditions. Both FD and ESRD are chronic conditions that impose a significant physical and emotional burden. Both are associated with high levels of psychological stress and an increased risk of developing psychopathological symptoms, such as anxiety and depression.

ESRD patients often require intensive therapies, such as dialysis, which is a challenging aspect of medical management, comparable to the intensive treatment needed for advanced FD.

Comparing Fabry patients with ESRD patients helps to isolate the psychological and behavioral effects specific to FD. This can identify differences that are unique to the disease rather than general factors associated with chronic illness.

Previous studies have suggested that chronic diseases, especially those involving renal function, significantly impact quality of life and psychological profiles. This comparison enables a deeper understanding of the unique characteristics of FD patients. The comparison between FD and ESRD is supported by the literature emphasizing shared clinical and psychological challenges in chronic diseases, particularly those affecting renal function.

Studies have highlighted that renal involvement in FD leads to progressive kidney damage, which is often comparable to the pathophysiological challenges faced by ESRD patients. For example, Monte et al. (27) emphasize the importance of multidisciplinary approaches to assess Fabry-related renal dysfunction and its psychological implications. Research by De Pasquale et al. (26) and others has shown that patients with ESRD undergoing dialysis often experience psychological distress, including anxiety, depression, and interpersonal sensitivity, which are also prominent in FD patients. Crosbie et al. (14) noted significant psychological distress in FD patients, with traits such as depression, anxiety, and obsessive-compulsiveness, paralleling findings in ESRD patients where the burden of chronic illness impacts their quality of life. Other studies suggest that maladaptive personality traits, such as schizoid and depressive characteristics, are more prevalent in FD patients compared with ESRD patients. This is supported by systematic reviews such as those conducted by Mroczek et al. (24), which discuss the neuropsychiatric and behavioral manifestations in FD patients, often comparing them to other chronic disease groups. Since both conditions involve significant renal impairment, the psychological and clinical burden of managing kidney disease provides a relevant framework for comparative studies, such as those examining personality traits and coping mechanisms.

Given these considerations, this study hypothesizes that FD patients may exhibit distinct psychological and personality profiles compared with ESRD patients. Specifically, we hypothesize that:

1. Personality Differences: FD patients will show higher levels of depressive, schizoid, and negativistic personality traits compared with ESRD patients. These traits are hypothesized to stem from the multisystemic impact and unique psychosocial burden of FD, such as chronic pain, stigma, and isolation.
2. Psychopathological Symptoms: FD patients will exhibit greater levels of somatization, anxiety, interpersonal sensitivity, and depression than ESRD patients. This is based on the literature suggesting that the unpredictable and progressive nature of FD exacerbates emotional distress.
3. Comparative Perspective: While both groups face chronic health challenges, the nature and focus of their burdens differ. ESRD patients’ psychological profiles may reflect the acute stressors of dialysis and treatment dependency, whereas FD patients’ profiles may be shaped by their systemic condition’s pervasive impact on their identity and social functioning.

Our study is the first to compare these two chronic diseases. The comparison between the personality and psychopathology of patients with FD and patients with ESRD could highlight significant differences in the psychological experiences associated with these conditions, offering important insights for the adjustment of psychoeducational treatments, psychotherapeutic support and for the improvement of psychological intervention in both pathologies. This study could contribute to a deeper understanding of the psychological factors that influence the adaptation and quality of life of patients, paving the way for more effective therapeutic interventions. In the following sections, we will discuss whether these hypotheses are supported by our findings, focusing on differences in personality traits and psychopathological symptoms.

## 2 Methods

### 2.1 Participants and procedure

Given that the present study is the first to describe whether Fabry patients vs. ESRD patients are characterized by specific personality characteristics and psychopathological symptoms, there was no related effect size to choose from for formal power analysis. The present study was conducted as a first hypothesis testing and should be used to design larger confirmatory studies.

This study enrolled 18 adult (age > 18 years) patients (14 females and 4 males, mean age 46.56) who had a genetically confirmed FD after a careful and standardized evaluation: FD diagnosis was performed with the genetic analysis for pathogenic *GLA* variants analyzed through the polymerase chain reaction in all participants from a peripheral blood sample. Analyses of the *GLA* gene, lyso-Gb3 levels, and α-Gal-A enzymatic activity were performed at Centogene^©^ Laboratories (Rostok, Germany) (26).

Of the 18 participants, 4 had the classic form (CL) of FD, 10 had the late onset form (LO) of FD, and 4 had the VUS (variant of uncertain significance) form of FD.

Regarding treatment, 4 patients were on enzyme replacement therapy (ERT) and 3 patients were on Chaperone Therapy.

Regarding medical comorbidity, all patients had impaired renal function, additionally 4 patients had hypertension and 4 patients had hypertrophic cardiomyopathy.

No patient had a history of psychiatric comorbidities.

#### 2.1.1 Recruitment

Patients were recruited at the Multidisciplinary Research Center for the Diagnosis and Treatment of Fabry Disease and Organ Transplantation of the University of Catania between April 2023 and September 2023.

At the enrollment date of the study, the total number of patients in the center was 54 (12 males and 42 females). All patients were contacted and asked to participate. Only 18 of the 54 patients contacted agreed to participate in our study. The rest of contacted patients preferred not to undergo the psychological-psychiatric evaluation.

Despite the male prevalence of FD documented in the literature, in our center a female prevalence was found. Moreover, women were the ones who showed greater willingness and interest in participating. Therefore, our sample consists of 14 females and 4 males.

Patients with low levels of education, < 8 years, (the MCMI requires a minimum of 8 years of schooling), psychiatric diagnoses, such as Alzheimer’s disease, mental retardation and other cognitive disorders, as they could compromise the ability to understand the items, and taking psychotropic drugs (antipsychotics and/or antidepressants) were excluded from the analysis.

#### 2.1.2 Final sample

Fabry patients (n=18) were compared with patients with ESRD (n=18) on renal replacement therapy (dialysis). FD patients, on average, had been diagnosed with the disease 4.94 years before, while patients with ESRD, on average, had been diagnosed with the disease 7.94 before (**Table 1**). Fabry patients had a microalbuminuria value (mean, mg/24 h) of 200.34 ± 51.3 and proteinuria (mean, mg/24 h) of 413 ± 85.4, indicative of impaired renal function.

**Table 1:** Descriptive statistics and group differences.

	Study variables	Fabry patients (n = 18)	Controls (n = 18)	Group differences
		N(%)/mean (sd, skewness)		Test, *p*-value
	Gender (F/M)	14/4	6/12	z = -2.57, *p* = .01**
	Age (years)	46.56 (14.80, 0.34)	41.33 (11.05, 0.34)	t = 1.2, *p* = .24
	Years of Education	12.39 (4.10, -0.07)	12.17 (3.54, 0.21)	t = 0.2, *p* = .86
Occupation	Employed	11 (61.11%)	9 (50.0%)	*X^2^ =* 5.1, *p* = .28
	Unemployed	6 (33.33%)	4 (22.22%)	
	Homemaker	0	2 (11.11%)	
	Student	0	1 (5.56%)	
	Retired	1 (5.56%)	2 (11.11%)	
Marital Status	Married	13 (72.2%)	12 (66.7%)	*X^2^ =* 3.83, *p* = .28
	Single	3 (16.7%)	6 (33.3%)	
	Divorced	1 (5.7%)	0	
	Widowed	1 (5.7%)	0	
	Years since diagnosis	4.94 (3.13, 2.25)	7.94(9.61, 1.47)	t = -1.26, *p* = .22
Personality Disorder Scales of the MCMI-III	Schizoid	63.78 (23.06, 0.17)	44.33 (18.15, -0.08)	^§^F = 8.11, *p* = .01**
	Avoidant	43.94 (27.84, -0.36)	30.44 (22.21, 0.47)	^§^F = 2.66, *p* = .11
	Dependent	42.06 (25.00, -0.41)	29.61 (21.66, 0.75)	^§^F = 2.51, *p* = .12
	Histrionic	63.44 (23.13, -0.52)	73.89 (21.88, 0.21)	^§^F = 1.88, *p* = .18
	Narcissistic	71.00 (16.07, 0.67)	74.83 (15.68, 0.75)	^§^F = 0.55, *p* = .46
	Antisocial	42.89 (22.55, -0.69)	34.44 (22.47, 0.15)	^§^F = 1.23, *p* = .28
	Aggressive (Sadistic)	48.28 (26.92, -0.30)	32.17 (23.01, 0.04)	^§^F = 3.62, *p* = .07
	Compulsive	81.22 (21.03, -0.70)	82.56 (21.02, 0.06)	^§^F = 0.04, *p* = .85
	Passive-Aggressive (Negativistic)	56.11 (22.65, -0.27)	38.67 (20.21, 0.37)	^§^F = 5.85, *p* = .02*
	Masochistic (Self-Defeating)	28.67 (23.32, 0.06)	25.22 (22.54, 0.23)	^§^F = 0.21, *p* = .65
	Schizotypal	39.22 (32.88, 0.12)	33.22 (30.05, 0.00)	^§^F = 0.36, *p* = .55
	Borderline	35.06 (22.61, 0.41)	20.94 (21.36, 0.93)	^§^F = 3.86, *p* = .06
	Paranoid	53.11 (23.03, -0.68)	41.33 (33.76, 0.34)	^§^F = 1.45, *p* = .24
	Depressive	40.78 (26.99, 0.15)	22.67 (25.05, 1.03)	^§^F = 4.96, *p* = .03*
SCL-90-R	Somatization (SOM)	1.12 (0.88, 0.49)	0.50 (0.44, 0.39)	^§^F = 7.68, *p* = .01**
	Obsessive-compulsive (O-C)	1.09 (0.85, 1.06)	0.55 (0.52, 1.04)	^§^F = 5.90, *p* = .02*
	Interpersonal sensitivity (I-S)	0.78 (0.68, 0.88)	0.25 (0.37, 2.62)	^§^F = 9.21, *p* = .01**
	Depression (DEP),	1.10 (0.74, 0.48)	0.45 (0.49, 1.64)	^§^F = 12.03, *p* = .002**
	Anxiety (ANX)	1.12 (0.71, 0.57)	0.49 (0.58, 0.81)	^§^F = 9.04, *p* = .01**
	Hostility (HOS)	0.67 (0.65, 1.46)	0.39 (0.49, 1.90)	^§^F = 2.40, *p* = .13
	Paranoid ideation (PAR)	0.76 (0.63, 0.95)	0.49 (0.67, 1.86)	^§^F = 1.60, *p* = .22
	Psychoticism (PSY)	0.68 (0.64, 1.30)	0.24 (0.28, 1.31)	^§^F = 7.51, *p* = .01**

Controls represents a group of individuals with end-stage renal disease (ESRD); ^§^represents group differences while controlling for gender; **represents *p* value <.01; *represents *p* value <.05; MCMI-III represents the Millon Clinical Multiaxial Inventory III; and SCL-90-R represents the Symptom Checklist-90-R.

As reported in **Table 1**, the two groups differed significantly only on gender distribution.

They were comparable for age, years of education, occupation, marital status, and years since diagnosis.

#### 2.1.3 Measures

All patients underwent a full psychological and psychiatric evaluation and were included in the study if they completed the Millon Clinical Multiaxial Inventory III and the Symptom Checklist-90-R (SCL-90 R).

The Millon Clinical Multiaxial Inventory III (MCMI-III) (28) Italian version by Zennaro et al. (29) was used to assess personality disorders. The MCMI-III is a psychological assessment tool intended to provide information on personality prototypes, characterological patterns, and behaviors to be explored clinically. The MCMI differs from other personality tests in that it is based on theory and is organized according to a multiaxial format. It is composed of 175 true-false questions represented by several scales: 14 personality disorder scales, 10 clinical syndrome scales, 5 correction scales (3 modifying indices: X, disclosure scale; Y, desirability scale; Z, debasement scale); 2 random response indicators, and 42 Grossman personality facet scales (based on Seth Grossman’s theories of personality and psychopathology). This study included only the 14 MCMI–III scales pertaining to personality disorders. Scores greater than 75 indicate the prevalence of the disorder, scores greater than 85 indicate a greater clinical severity of the disorder (29).

In our sample, Cronbach’s alpha for each personality disorders scale was acceptable (Schizoid: α = 0.76; Avoidant: α = 0.78; Dependent: α = 0.74; Histrionic: α = 0.68; Narcissistic: α = 0.77; Antisocial: α = 0.74; Aggressive (Sadistic): α = 0.84; Compulsive: α = 0.71; Passive-Aggressive (Negativistic): α = 0.77; Masochistic (Self-Defeating): α = 0.80; Schizotypal: α = 0.90; Borderline: α = 0.77; Paranoid: α = 0.87; Depressive: α = 0.86).

Regarding the clinical syndromes evaluated with the MCMI-III, we decided not to consider them in the analyses, as the psychopathological symptoms were already completely evaluated with the SCL-90 R, and therefore we decided, with the suggestion of a methodology expert, that simplifying the variables would have been more appropriate, also given the small sample size.

The SCL-90-R (30) Italian version by Prunas et al. (31) was used to assess a broad range of psychological problems and symptoms of psychopathology. The SCL-90-R consists of 90 items represented by nine primary symptom dimensions: somatization (SOM), obsessive-compulsive (O-C), interpersonal sensitivity (I-S), depression (DEP), anxiety (ANX), hostility (HOS), phobic anxiety (PHOB), paranoid ideation (PAR), psychoticism (PSY). Scores equal to or greater than 1 are clinically significant (30). The PST (Positive Symptom Total) is one of the indices derived from the SCL-90 questionnaire. It refers to the total score of the items that indicate the presence of symptoms perceived as problematic by the patient. The PST can be used in combination with other scores derived from the SCL-90 (such as the PSDI - Positive Symptom Distress Index) to obtain a complete picture of the psychopathological symptomatology, both in terms of frequency and intensity of symptoms. In our sample, Cronbach’s apha was acceptable for each dimension (SOM: α = 0.85; O-C: α = 0.86; I-S: α = 0.82; DEP: α = 0.85; ANX: α = 0.86; HOS: α = 0.70; PAR: α = 0.73; PSY: α = 0.77) with the exception of phobic anxiety (PHOB: α = 0.52) that was not included in the analyses.

### 2.2 Statistical analysis

Analyses were performed using R software (32). Differences between groups on sociodemographic variables were analyzed using chi-square (categorical variables) or t-tests (continuous variables). As the groups differed significantly in gender distribution, with a higher number of males in controls (**Table 1**), gender was included as a covariate in all analyses.

Linear model analysis, considering group (patients with FD, patients with ESRD) as predictor, was performed to compare personality characteristics (schizoid, avoidant, dependent, histrionic, narcissistic, antisocial, aggressive (Sadistic), compulsive, passive-aggressive (negativistic), masochistic (self-defeating), schizotypal, borderline, paranoid, depressive) and symptoms of psychopathology (somatization, obsessive-compulsive, interpersonal sensitivity, depression, anxiety, hostility, paranoid ideation, and psychoticism) between groups. Residual plots were used to evaluate the normality and homogeneity of the variance. The scatterplot of the standardized residuals showed that the data met the assumptions of homogeneity of variance and linearity. Effect sizes are reported in terms of Cohen’s d, with a d ≥ 0.5 indicating a moderate or greater effect size and a d ≥0.8 indicating a large effect size (33).

## 3 Results

Descriptive statistics by group and main results are reported in **Table 1**.

**Figure 1:**
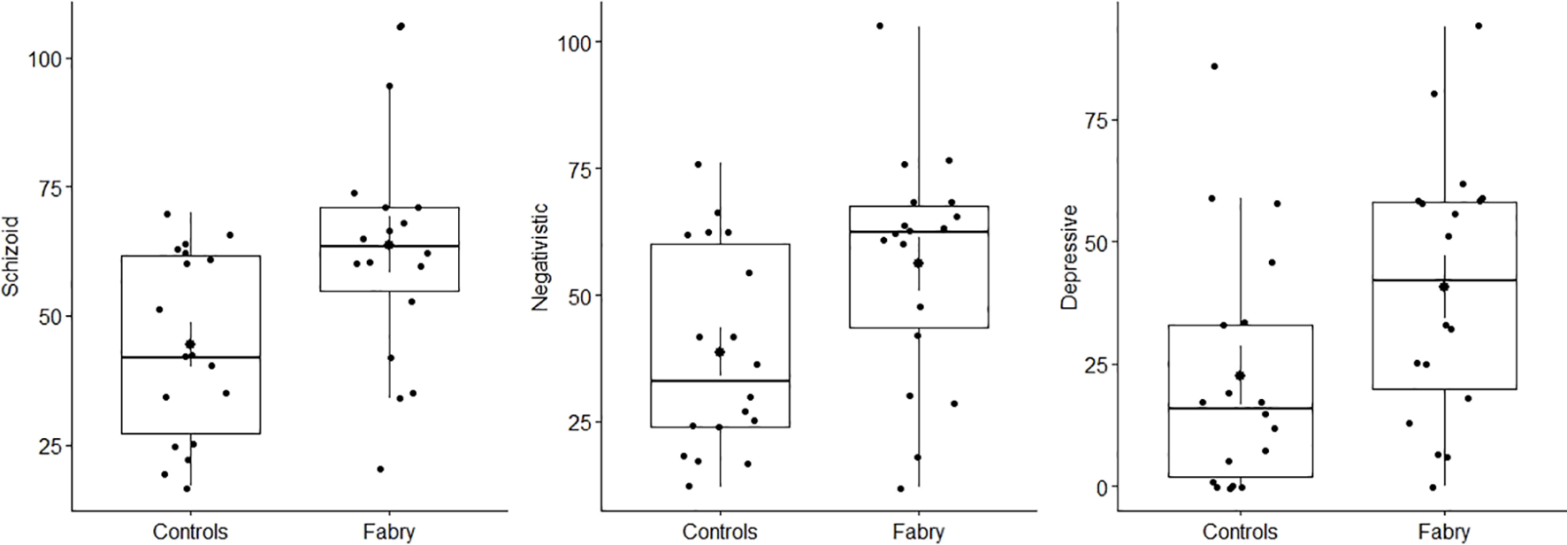
Group differences in schizoid, negativistic, and depressive scales of the personality disorder scales of the MCMI-III. Fabry represents Fabry patients and Controls represents the group of individuals with end-stage renal disease (ESRD).

As reported in **Table 1**, groups differed significantly on three of the personality disorder scales of the MCMI-III. Specifically, patients with FD scored higher on schizoid (Cohen’s d = 0.94), negativistic (Cohen’s d = 0.81), and depressive scales (Cohen’s d = 0.70) than controls (patients with ESRD. See **Figure 1**). The significant difference in gender distribution between the FD and ESRD groups was noted in our study. Fabry Disease (FD) group: 14 females and 4 males; End-Stage Renal Disease (ESRD) group: 6 females and 12 males. This resulted in a statistically significant difference in gender distribution (z = -2.57, p = .01). To address this imbalance, gender was included as a covariate in all analyses to account for its potential influence on the results. This ensures that the observed differences between the groups in personality and psychopathology are not confounded by gender effects.

Of note, the same results are also obtained by using non-parametric tests (see the **Supplementary Material**).

**Figure 2:**
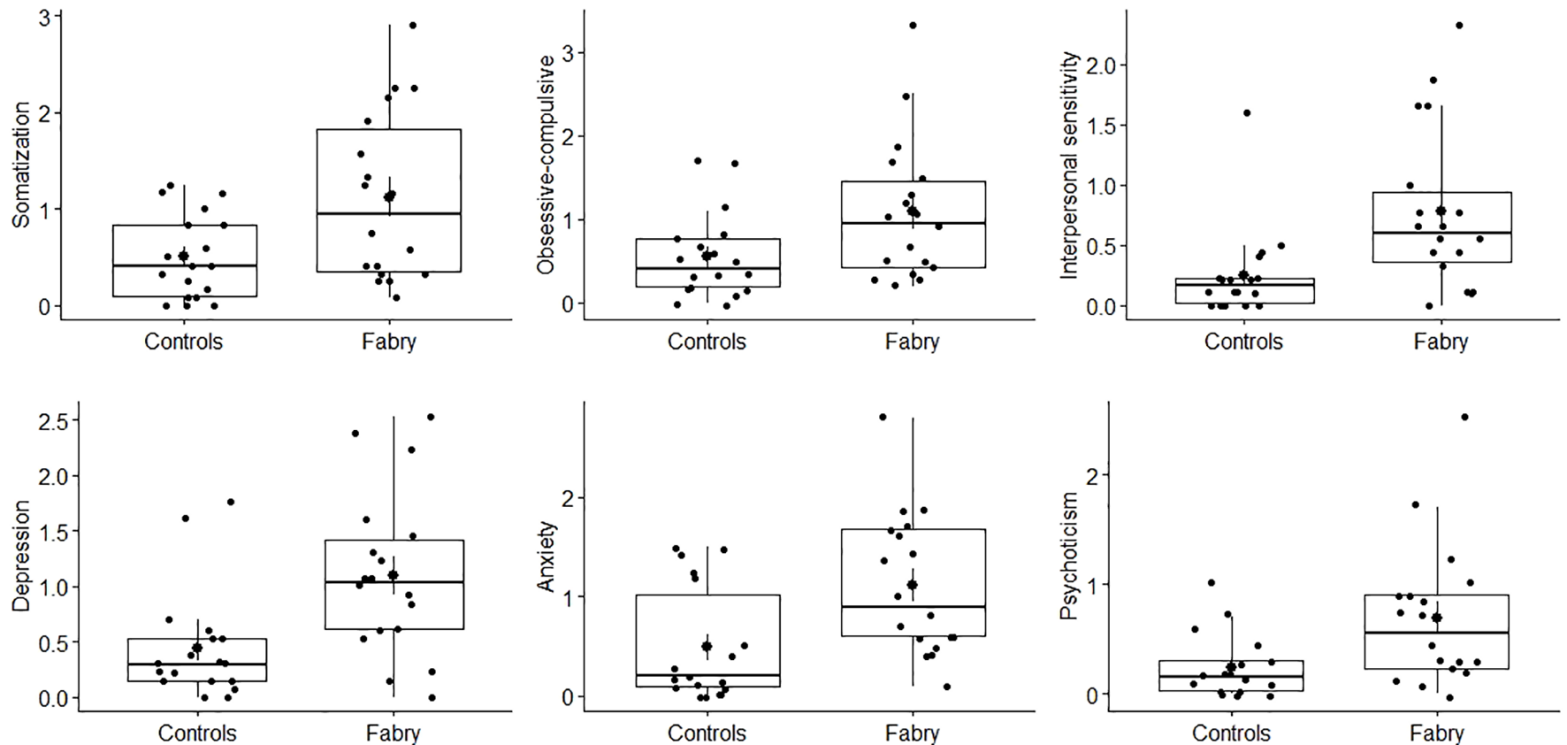
Group differences in somatization, obsessive-compulsive, interpersonal sensitivity, depression, anxiety, and psychoticism dimensions of the SCL-90-R. Fabry represents Fabry patients and Controls represents the group of individuals with end-stage renal disease (ESRD).

Groups also differed significantly on six of the SCL-90-R dimensions. Specifically, patients with FD scored higher on somatization (Cohen’s d = 0.89), obsessive-compulsive (Cohen’s d = 0.77), interpersonal sensitivity (Cohen’s d = 0.97), depression (Cohen’s d = 1.04), anxiety (Cohen’s d = 0.97), and psychoticism (Cohen’s d = 0.90) than controls (See **Figure 2**).

## 4 Discussion

Few studies have explored personality and psychological functions in FD patients, but research on chronic diseases in general suggests higher scores on personality scales compared with normative samples.

Studies on many chronic diseases indicate an impact of personality traits on mental health and quality of life (22, 23, 34).

The literature, albeit limited, highlights how patients with FD are at higher risk of developing psychological distress, psychopathology, and impaired quality of life. Regarding the FD patients, a recent systematic review by Mroczek et al. (24) highlighted specific personality characteristics such as avoidant and antisocial personality, and aggressive behavior. Similar personality characteristics were also found in the dialysis patients with ESRD; specifically, patients appeared indifferent to the needs of others, basically anxious and emotionally unstable (25).

The results of our preliminary study revealed significantly higher levels of schizoid, depressive, and negativistic personological constructs in FD than ESDR patients.

### 4.1 Schizoid personality and FD

Schizoid personality is characterized by marked social isolation, disinterest in interpersonal relationships, and a preference for solitude. Patients with Fabry disease, facing a debilitating condition that can limit their daily and social activities, may develop schizoid traits as a coping mechanism to the disease. Social isolation may be a consequence of both physical fatigue and the feeling of not being understood or being stigmatized because of the rare disease. In addition, the disease may favor the tendency to avoid social interactions or to minimize one’s emotional needs, in order to avoid further discomfort (26).

### 4.2 Depressive personality and FD

A depressive personality construct fits into a dimensionalized framework of assessing psychopathology. In the depressive personality trait there is a strong sense of loss, of renunciation and there is a lack of hope of being able to experience a sense of joy again. The depressed personality trait experiences permanent pain while pleasure is an experience that is conceived as impossible. Moreover, it could be anticipated that Depressive Personality would be represented by elevations in other DSM-5 personality traits, including hostility, restricted affectivity, withdrawal, intimacy avoidance and rigid perfectionism, aspects that refer to the schizoid and negativistic personality (21).

### 4.3 Negativistic personality and FD

Negativistic personality is characterized by a resistance to following norms and expectations, frequently associated with an attitude of distrust towards others and institutions. In FD patients, this characteristic may derive from a long history of frustration due to the difficulty in diagnosing the disease and receiving adequate treatment, as well as from the experience of being ignored or misunderstood. In addition, FD has a progression that may involve an experience of constant “fighting” against the symptoms, leading to the formation of an attitude of opposition or distrust towards medicine or therapeutic recommendations (35).

In line with findings in the literature on this topic, we also found higher levels of psychopathological symptoms in FD patients than controls (patients with ESDR).

Furthermore, the PST of the SCL-90 of these subjects is equal to 45.16, indicating a relatively high level of psychopathological symptoms perceived by the subject. This suggests the presence of significant psychological symptoms or difficulty in managing a series of emotional and psychological problems.

Specifically, significantly higher scores were highlighted in the dimensions of somatization, obsession-compulsion, interpersonal sensitivity, depression, anxiety, and psychoticism.

The psychopathological traits that we have highlighted in our study of patients with FD are extremely relevant to understanding the psychological and psychiatric effects of this rare condition. Chronic diseases, such as FD, can involve a series of psychological and psychosocial challenges that profoundly affect the mental well-being of patients. The traits that emerged are known to be frequently associated with debilitating chronic diseases, both directly, through chronic physical pain, and indirectly, due to social isolation and difficulty in treatment (36).

### 4.4 Somatization and FD

Somatization is a common response to chronic and debilitating diseases. FD patients may develop physical symptoms that are not only a direct manifestation of the disease, but also a form of expression of emotional distress. The difficulty in diagnosing and treating the disease adequately may lead patients to focus on physical symptoms and have difficulty expressing their psychological distress. Chronic pain, which is a central feature of FD, may be a major cause of somatization, where physical symptoms intertwine with emotional ones (37).

### 4.5 Obsessive-compulsive traits and FD

The presence of obsessive-compulsive traits in patients with FD may be a response to the need for control in a context of uncertainty and frustration. Rigid control and ritualizations may be an attempt to manage the unpredictability and difficulty in managing the disease. Furthermore, the focus on health and therapies may reinforce obsessive behaviors (38). Frustration resulting from the complexity of the disease and treatments may intensify the need to establish reassuring routines.

### 4.6 Interpersonal sensitivity and FD

High interpersonal sensitivity is a characteristic that can emerge when patients feel vulnerable due to their disease and its visibility (or invisibility) in social relationships. The inability to fully understand their health status and the experience of the rare disease can lead to the perception of strangeness or incomprehension by others, intensifying emotional reactivity in social interactions. Studies suggest that patients with rare diseases tend to develop greater emotional sensitivity due to the difficulties in making their condition understood by others (39).

### 4.7 Depression and anxiety in FD

Depression and anxiety are common in patients with chronic diseases, including rare ones such as FD. The daily struggle with physical symptoms, difficulties in coping with chronic pain, and frustration with disease management are factors that contribute to an increased risk of mood and anxiety disorders. Uncertainties about the duration of the disease and its psychological complications (such as difficulty integrating into social and work life) may increase the risk of developing these psychopathological traits (34, 37). Furthermore, the impact of the disease on quality of life is an important risk factor for the development of anxiety and depression disorders (40).

### 4.8 Psychoticism and FD

Psychoticism, a trait associated with dissociative, paranoid, or bizarre thoughts and behaviors, may reflect a detachment from reality in response to a chronically anxious and stressful life. The experience of rare disease and the difficulty in finding effective treatment may lead to distortions in thinking and in the way of perceiving oneself and others, intensifying the risk of developing psychotic traits. Patients with chronic diseases may develop a distorted view of reality as a defense mechanism against psychological pain (41).

It can be hypothesized that these predominant symptoms could represent a maladaptive response to the disease condition of FD patients, whose depressive, schizoid and negativistic personality characteristics appear to be connected. One possible explanation for the elevated personality traits in FD patients is the early onset of the disease and its diagnosis during formative developmental years. Early diagnosis may shape personality development by exposing individuals to chronic stress, pain, and stigma during critical periods of psychosocial growth. These stressors might foster traits such as withdrawal, negativism, or depressive tendencies as coping mechanisms against the uncertainty and isolation associated with the disease.

Additionally, it is plausible that some personality traits predate the diagnosis of FD and represent a pre-existing vulnerability. Chronic illness could then exacerbate these traits, creating a feedback loop in which the disease and psychological characteristics amplify each other. For example, individuals with higher baseline sensitivity or depressive tendencies might struggle more with the challenges posed by FD, leading to heightened levels of these traits over time.

The pervasive nature of FD, which affects multiple organ systems, imposes unique psychological burdens compared with ESRD.

### 4.9 The link between personality and psychopathology in FD

Based on the results of this study, the authors believe that individuals with FD need an assessment that studies the personological and emotional profiles, and possible psychopathologies. The in-depth study of the FD patient’s personality is necessary as the personological characteristics modify the degree of awareness and acceptance of a chronic illness with a strong emotional burden and influence the possible manifestation of psychopathology of varying degrees up to actual mental disorders (42).

These findings are important in the prognosis of the disorder, as identifying maladaptive personality patterns and treating the psychopathological symptoms that emerge can improve treatment adherence, facilitate the adoption of effective coping strategies, prevent self-harming behavior and provide a basis for psychotherapeutic interventions targets, contributing to a more complete and integrated therapeutic approach.

FD, being a rare genetic condition, presents with diverse symptoms, including chronic pain, fatigue, and psychosocial impacts. The future of psychological care for FD patients could benefit from personalized therapy approaches. Rather than a one-size-fits-all intervention, psychological treatment could be more effective if adapted to the individual’s specific coping mechanisms, pain levels, and emotional responses. This could involve regular assessments of patients’ psychological states and ongoing adjustments to interventions such as Cognitive Behavioral Therapy (CBT) or mindfulness-based stress reduction (42).

Literature data recommends the need for psychological counseling interventions, psychotherapy and prevention programs for the management of psychological distress, the emotional burden of the disease and possible psychopathology in FD patients (1, 40).

### 4.10 The importance of a multidisciplinary approach

Since FD is a serious and complex pathology with systemic involvement, the approach to this disease is complex and necessarily multidisciplinary (43). The presence of a multidisciplinary team is fundamental in terms of diagnosis, management and treatment of patients with FD to plan treatment paths that are as integrated as possible, including psychotherapy and pharmacotherapy in the management of these patients. A multidisciplinary approach in FD is essential to address the complexity of the disease, improve the quality of care and optimize clinical outcomes. This type of approach guarantees complete and integrated management of the disease, addressing not only the medical aspects but also the psychological and social dimensions, providing the patient with the best possible support (44).

### 4.11 The limitations of our study

In our study some limitations must be considered: the small sample size, the cross-sectional nature of the design, the use of self-report measures, and the gender imbalance among groups. Moreover, the FD patient cohort manifests a distinct renal functional impairment compared with the control group. It is advisable to compare two patient cohorts characterized by an equivalent degree of renal insufficiency—one with FD and another without FD.

The distribution of 42 females and 12 males among the 54 patients with FD in our center, and consequently the 14 female and 4 male participants in our study, is certainly atypical, considering that FD is traditionally more prevalent in males due to its X-linked transmission. However, mild forms of FD are more common in female carriers, who may not develop severe symptoms or may manifest them in a very mild or delayed manner. The disease may have been diagnosed in these patients because of more subtle symptoms, or with a later diagnosis, such as pain, renal or cardiovascular problems that were not immediately recognized as related to FD. Since the severity of the disease in women can vary widely, it is possible that our center has a high percentage of female patients with less severe forms, but still diagnosed thanks to genetic tests or clinical suspicion.

Some studies support the idea that the higher prevalence of females in our center could be due to a combination of factors, including the diagnosis of milder forms in the female population and the effect of family screening, which can lead to a greater number of women identified, even in the absence of severe symptoms (45–47).

Nevertheless, despite the preliminary nature of our findings, they bear noteworthy clinical significance. The results of our study provide information and some significant evidence to support the hypothesis that the psychological distress of FD patients could be due to the combination of several factors, including some dysfunctional personality traits, psychopathology and perceived quality of life. The findings and limitations of the present study will be used in future work to design new studies with larger samples to provide further evidence of the connection between personality, emotional profile and quality of life. Understanding the unique psychological profiles of FD patients highlights the need for tailored interventions. Early psychological support focusing on building resilience and adaptive coping strategies may mitigate the development of maladaptive personality traits and psychopathological symptoms. Interventions such as cognitive-behavioral therapy (CBT), mindfulness-based approaches, and support groups could address the specific challenges of FD patients, including isolation, stigma, and emotional distress (48–52).

## 5 Conclusions

The diagnosis of a rare disease such as FD can be both upsetting and stressful, often leading to feelings of anxiety, depression, and uncertainty about the future. Psychological evaluation in FD is essential to address the emotional, cognitive, and behavioral aspects of the disease. Given the progressive nature of FD, continuous psychological monitoring is crucial to adapt management strategies according to the evolution of the disease and its symptoms, ultimately improving the quality of life for both the patient and their family. Additionally, the importance of an integrated approach cannot be overstated. A multidisciplinary team is essential for planning treatment paths that are as comprehensive as possible, incorporating psychotherapy and pharmacotherapy to ensure holistic management of the disease. Future research should explore the developmental trajectory of personality traits in FD patients, investigating whether these traits are primarily shaped by the disease or reflect pre-existing vulnerabilities. Longitudinal studies with larger sample sizes and comparisons across other chronic conditions would provide deeper insights into these dynamics.

## Data Availability

The raw data supporting the conclusions of this article will be made available by the authors, without undue reservation.
