# Supplementary material for: Comparison of personality and psychopathology in patients with Fabry disease and patients with end-stage renal disease: a preliminary study
Source: Front Psychiatry. 2025 Feb 27;16:1460260. doi: 10.3389/fpsyt.2025.1460260 (PMC11903431; doi:10.3389/fpsyt.2025.1460260)
Supplement: Supplementary file 1 [file Table1.docx]

Independent 2-group Mann-Whitney U Test

data: MCMI_Schizoid by Group

W = 84, p-value = 0.01

data: MCMI_Avoidant by Group

W = 118, p-value = 0.2

data: MCMI_Depressive by Group

W = 97, p-value = 0.04

data: MCMI_Dependent by Group

W = 112, p-value = 0.1

data: MCMI_Histrionic by Group

W = 198, p-value = 0.3

data: MCMI_Narcissistic by Group

W = 181, p-value = 0.6

data: MCMI_Antisocial by Group

W = 136, p-value = 0.4

data: MCMI_Aggressive by Group

W = 102, p-value = 0.06

data: MCMI_Compulsive by Group

W = 164, p-value = 1

data: MCMI_Negativistic by Group

W = 88, p-value = 0.02

data: MCMI_Masochistic by Group

W = 150, p-value = 0.7

data: MCMI_Schizotypal by Group

W = 150, p-value = 0.7

data: MCMI_Borderline by Group

W = 100, p-value = 0.05

data: MCMI_Paranoid by Group

W = 115, p-value = 0.1

data: SCL_SOM by Group

W = 92, p-value = 0.03

data: SCL_OC by Group

W = 90, p-value = 0.02

data: SCL_I-S by Group

W = 65, p-value = 0.002

data: SCL_DEP by Group

W = 71, p-value = 0.004

data: SCL_ANX by Group

W = 66, p-value = 0.002

data: SCL_HOS by Group

W = 110, p-value = 0.1

data: SCL_PHOB by Group

W = 144, p-value = 0.6

data: SCL_PAR by Group

W = 105, p-value = 0.07

data: SCL_PSY by Group

W = 78, p-value = 0.007
